# Effects of dietary PQQ·Na_2_ level on performance and bone microstructure of aged laying hens

**DOI:** 10.1016/j.psj.2026.107421

**Published:** 2026-07-12

**Authors:** M. Feng, Y. Tang, Q. Huang, M. Lu, H. Zeng, Y. Bai, J. Lu

**Affiliations:** a Institute of Feed Science, College of Animal Sciences, Zhejiang University, Hangzhou, 310058, China; b ZJU-Xinchang Joint Innovation Centre (TianMu Laboratory), Gaochuang Hi-Tech Park, Xinchang, 312500, China; c Zhejiang Medicine Co., Ltd., Shaoxin, 312355, China

**Keywords:** Aged laying hen, Cecal microbiota, Calcium and phosphorus metabolism, Pyrroloquinoline quinone disodium salt, Production performance

## Abstract

A total of 480 Jinghong No. 1 laying hens (400 d of age) were randomly assigned to five groups for a 7-week feeding trial: a control group and four treatment groups supplemented with 0.5, 1.0, 1.5, or 2.0 mg/kg pyrroloquinoline quinone disodium salt (PQQ·Na_2_), respectively. This study evaluated the effects of dietary PQQ·Na_2_ on production performance, egg quality, calcium and phosphorus metabolism, tibial microstructure, intestinal morphology, and cecal microbiota. Dietary supplementation with 0.5 mg/kg PQQ·Na_2_ significantly increased laying rate, eggshell strength, and albumen height (*P* < 0.05). Serum alkaline phosphatase, 25-hydroxyvitamin D_3_, osteocalcin, and calcium concentrations were increased, whereas calcitonin and parathyroid hormone concentrations were decreased (*P* < 0.05). Micro-CT analysis showed that the 0.5 mg/kg PQQ·Na_2_ treatment increased tibial bone mineral density and bone volume fraction and improved trabecular microstructure, although tibial calcium and phosphorus contents were not significantly changed. In addition, 16S rRNA sequencing indicated that PQQ·Na_2_ altered cecal microbial composition, including changes in the relative abundances of several dominant genera. Overall, supplementation with 0.5 mg/kg PQQ·Na_2_ improved laying performance, egg quality, and bone health in aged laying hens, potentially through coordinated regulation of calcium-phosphorus metabolism, intestinal morphology, tibial microstructure, and cecal microbiota.

## Introduction

Egg production in China is becoming increasingly intensive, and the laying cycle is being continuously extended. Consequently, hens in the late laying period (>50 weeks of age) face significant performance challenges, including declining egg production and deteriorating eggshell quality. According to industry statistics, the national monthly average inventory of laying hens in 2025 was approximately 1.34 billion birds. The decline in eggshell quality results in thinner eggshells, reduced shell strength, and an increased cracked egg rate, leading to substantial economic losses.

Imbalanced calcium and phosphorus ratios can directly cause eggshell quality deterioration in aged laying hens. A single egg contains approximately 2 g of calcium, representing about 10% of the total body calcium of a laying hen. This substantial allocation suggests that calcium transport and metabolic turnover are more rapid in laying hens than in other vertebrates (Hester, 2017). Modern commercial laying hens, with their high production efficiency, can lay up to 300 eggs per year, placing their calcium metabolism in a persistent state of “calcium turnover” (Sinclair-Black et al., 2023). Phosphorus, works in combination with calcium metabolism, plays a key role in energy metabolism and bone mineralization. Aged laying hens exhibit reduced intestinal calcium absorption efficiency (Wang et al., 2021), diminished renal calcium reabsorption capacity (Garcia-Mejia et al., 2024), and an imbalance between bone calcium mobilization and deposition, leading to an insufficient calcium absorption for eggshell formation and ultimately resulting in deteriorated eggshell quality. Therefore, nutritional strategies that enhance calcium and phosphorus utilization are urgently needed for aged laying hens.

Pyrroloquinoline quinone disodium (PQQ·Na_2_) has emerged as a promising nutritional intervention. Pyrroloquinoline quinone(PQQ), first discovered in 1979, was identified as a prosthetic group of bacterial dehydrogenases (Salisbury et al., 1979). PQQ exhibits significant biological functions, including antioxidant activity, growth promotion, neurotrophic effects, and regulation of mitochondrial biogenesis (Petricca et al., 2025; Aboulhassane et al., 2026). PQQ·Na_2_, the disodium salt form of PQQ, offers improved water solubility and stability, making it a common additive in current research and applications.

Shao et al. (2023) reported that dietary supplementation with PQQ·Na_2_ in aged laying hens increased albumen height and Haugh units while reducing malondialdehyde (MDA) content in the liver and yolk through activation of the nuclear factor erythroid 2-related factor 2 (Nrf2)/heme oxygenase-1 (HO-1) antioxidant pathway. (Shao et al., 2023). Similarly, Wang et al. (2018) found that supplementation with 0.08 mg/kg significantly increased plasma glutathione peroxidase (GSH-Px) activity and total antioxidant capacity (T-AOC), thereby enhancing liver antioxidant capacity in aged laying hens (Wang et al., 2018).

From a safety perspective, PQQ·Na_2_ has received regulatory approval for human consumption as a novel food ingredient in China (National Health Commission of the People's Republic of China, 2022) and the European Union (Turck et al., 2017; European Commission, 2018). This safety foundation supports its application in livestock and poultry production.

PQQ·Na_2_ promotes mitochondrial biogenesis and increases ATP production via the PGC-1α pathway (Chowanadisai et al., 2010), and scavenges mitochondrial reactive oxygen species to alleviate oxidative stress (Shao et al., 2023). However, it remains unclear whether this enhancement in energy metabolism can further drive active calcium (Ca) and phosphorus (P) transport and homeostatic regulation in aged laying hens. Intestinal and renal transcellular transport of Ca and P depends on ATP-dependent carriers such as Ca^2+^-ATPase and NaPi-IIb. Additionally, the eggshell gland deposits Ca at a high rate of 100-150 mg Ca/h. Both are energy-intensive processes highly sensitive to mitochondrial ATP production. Therefore, the cascade relationship between PQQ·Na_2_-improved energy metabolism and key regulators-including parathyroid hormone (PTH), calcitonin, vitamin D_3_ activation (25-OH-D_3_), and bone metabolism (bone mineral density and osteocalcin)—requires systematic investigation in aged laying hens.

Existing studies on PQQ·Na_2_ in aged laying hens have largely focused on the low-dose range of 0.04-0.40 mg/kg, targeting antioxidant function and production performance (Wang et al., 2018; Shao et al., 2023). To date, no study has reported the dose-response relationship of PQQ·Na_2_ within the range of 0.5-2.0 mg/kg regarding bone health, eggshell quality, and gut microbiota in aged laying hens. Preliminary experiments have tested doses up to 1.6 and 3.2 mg/kg without adverse effects (Shao et al., 2023), indicating that the selected dose range (0.5-2.0 mg/kg) is within a safe window.

Accordingly, this study used aged laying hens as a model to evaluate the ameliorative effects of dietary supplementation with four doses of PQQ·Na_2_ (0.5, 1.0, 1.5, and 2.0 mg/kg) on osteoporosis and to elucidate the underlying mechanisms. The findings will provide theoretical support for the application of PQQ·Na_2_ in laying hen production and offer a novel nutritional strategy for improving eggshell quality and alleviating osteoporosis in aged laying hens.

## Materials and methods

### Experimental design and diets

This study used a completely randomized design (CRD). A total of 480 healthy Jinghong No. 1 laying hens (57 weeks old) with comparable initial body weights and laying rates were randomly assigned to five groups (one control group and four experimental groups) using a random number generator (Microsoft Excel, RAND function). Each group contained eight replicates of 12 hens each. The control group received a corn-soybean meal basal diet, while the experimental groups received the basal diet supplemented with 0.5, 1.0, 1.5, and 2.0 mg/kg PQQ·Na_2_ respectively. The composition and nutrient levels of the basal diet are shown in Table 1. The nutrient levels of the basal diet were formulated according to the Chicken Feeding Standard (NY/T 33-2004) and subsequently verified by actual measurement using national standard methods. Key nutritional indicators, including crude protein, calcium, total phosphorus, metabolizable energy, lysine, and methionine, were determined based on actual measured values; deviations between measured and calculated values were all less than 5%. The experiment included a 1-week adaptation period, during which daily laying rates were recorded. The formal experiment began when no significant differences in laying rate were observed among groups (*P* > 0.05) and lasted for seven weeks.

**Table 1:** Composition and nutrient levels of the basal diet (as-fed basis).

Items	Content
Ingredients	
Corn	60.15
Soybean meal (43% Crude Protein)	22.00
Soybean oil	2.00
Rapeseed meal	4.0
CaCO_3_	9.50
CaHPO_4_	1.60
DL-Met	0.10
L-Thr	0.05
NaHCO_3_	0.10
NaCl	0.30
Choline chloride (50%)	0.10
Vitamin and mineral premix ^1)^	0.10
Total	100.00
Nutrient levels ^2)^	
AME, MJ/ kg	11.40
Crude Protein	16.70
Ca	3.75
Available Phosphorus	0.35
D-Lys	0.77
D-Met	0.34
D-Thr	0.56
D-Trp	0.17

1) The premix provided the following per kg of the diet: VA 8 000 IU, VD3 2500 IU, VE 20 IU, VK_3_ 3.5 mg, VB_1_ 2 mg, VB_2_ 6.4 mg, VB_6_ 4 mg, VB_12_ 0.2 mg, D-biotin 11 mg, D-pantothenicacid 12mg, folic acid 1mg, nicotinamide 40 mg, Cu (CuSO_4_.5H_2_O) 8 mg, Fe (FeSO4. H_2_O) 60 mg, Mn (MnSO_4_·H_2_O) 60 mg, Zn (ZnSO_4_ ·H_2_O)80 mg, I (KI) 0.35 mg, Se (Na_2_ SeO_3_) 0.3 mg.

2) Crude Protein was a measured value, while the other nutrient levels were calculated values

Sample size was calculated based on data from a similar study by Shao et al. (2023), using eggshell strength as the primary outcome (Shao et al., 2023). A moderate effect size (Cohen's d = 0.6) was assumed (Cohen, 2013), with α = 0.05 and a statistical power (1-β) of 0.80. Using G*Power 3.1 software, the minimum sample size required per treatment for terminal sampling was calculated to be 8 hens. To account for the monitoring of production performance throughout the trial and to meet the terminal sampling requirement, the study was designed with 5 treatments × 8 replicates × 12 hens (total 480 hens). At the end of the experiment, one hen per replicate was randomly selected for terminal sampling, providing 8 hens per treatment for core indicator analysis, thereby meeting the calculated statistical power requirement. This sample size also complies with the Chinese National Standard "Feed Safety Evaluation - Layer Feeding Trial Technical Specification" (GB/T 40837-2021). PQQ·Na_2_ was purchased from Zhejiang Medicine Co., Ltd., and had a purity of 99.9%.

### Housing and management

The experiment was conducted in an environmentally controlled house at Kejia Poultry Industry Co., Ltd., in Xinchang, Zhejiang, China. The house was equipped with three-tier, H-type stacked cages, with six hens per cage, corresponding to a stocking density of 450 cm^2^ per bird. Ambient temperature and relative humidity were maintained at 20–23°C and 55%-65%, respectively. A photoperiod of 16 hours of light per day was provided at an intensity of 10 lux at bird-head level. Throughout the 7-week trial, hens had ad libitum access to feed and water. Feed was provided twice daily (08: 30 and 16: 30), and eggs were collected daily at 09: 00, according to the housing and management protocols described by Wan et al. (2023) and Lay et al. (2011) (Lay et al., 2011; Wan et al., 2023).

### Sample collection

On the day before the end of the trial, hens were fasted for 12 h with free access to water. On the final day, one hen per replicate was randomly selected (total 40 hens; 8 hens per group). After weighing, hens were euthanized by trained professionals using cervical dislocation in a quiet, dark environment. All procedures involving animal handling, care, and euthanasia were conducted in accordance with the principles and specific guidelines presented in the Guide for the Care and Use of Agricultural Animals in Research and Teaching (4th edition, 2020). No surgical procedures (including invasive procedures or caponization) were performed in this study; therefore, anesthetic agents were not required. Following euthanasia, fresh blood was collected into coagulation tubes, allowed to stand at room temperature for 1 h, and centrifuged at 3, 000 rpm for 10 min to obtain serum, which was aliquoted and stored at -80°C. The liver, oviduct, ovary, duodenum, jejunum, ileum, cecal contents, left and right tibiae, and femurs were collected and stored at -80°C for subsequent analysis.

### Ethics statement

All animal experimental procedures were based on a protocol approved by the Animal Care Advisory Committee of Zhejiang University (Approval No. ZJU20260328, Hangzhou, China). All birds used in this study were ethically handled. All animal operations during the experiment strictly complied with the Regulations for the Administration of Laboratory Animals of the People’s Republic of China (revised in 2017), Technical Specification for Laying Hen Feeding Trials for Safety Evaluation of Animal Feeds (GB/T 40837-2021), and relevant industry standards for laying hen management. Animal welfare was ensured, and to minimize animals suffering.

### Measurement indices and methods

#### Production performance

In the formal experimental period, egg production and egg weight were measured daily by replicate group. The laying rate and average egg weight were calculated. Residual feed was weighed at regular intervals each week, and the average daily feed intake per hen and the feed-to-egg ratio were calculated.

#### Egg quality

On days 20 and 49 of the experimental period, three eggs with weights close to the replicate average were randomly selected from each replicate. Egg quality parameters were measured using a DET-6000 digital egg tester (Nabel Co., Ltd., Kyoto, Japan), including albumen height (mm), yolk color (YolkFan™ color score: 1-16), and eggshell strength (N). The Haugh unit was calculated using the formula: HU =100 log(h-1.7w^{0.37}+7.6), where HU = Haugh unit, h = albumen height (mm), and w = egg weight (g). The egg shape index was calculated by measuring the long and short axes with a vernier caliper (long axis/short axis).

Day 20 (end of week 3) represents the initial manifestation of PQQ·Na_2_ effects after the adaptation period, reflecting short-term supplementation effects; day 49 (end of week 7) represents the end of the formal experimental period, reflecting long-term cumulative effects. These two time points align with the production performance statistical intervals (weeks 1 to 3, 3 to 7, and 1 to 7), allowing correlation analysis between dynamic changes in egg quality and laying performance. Additionally, this dual time-point design follows the approach used in previous studies (Shao et al., 2023),ensuring data comparability.

To minimize observer bias, all egg quality measurements were conducted by personnel blinded to the treatment groups. Samples were coded, and blinding was maintained until data compilation.

#### Serum biochemical parameters

Serum calcium, phosphorus, and alkaline phosphatase (ALP) activity were measured using commercial kits (calcium: Cat. No. HY-N0021; phosphorus: Cat. No. HY-N0022; ALP: Cat. No. HY-A0005; Beijing ZhongSheng BeiKong Biotechnology Co., Ltd., Beijing, China) on a Beckman AU480 automatic biochemical analyzer. Serum calcitonin (CT, Cat. No. HY-J0002), parathyroid hormone (PTH, Cat. No. HY-A0013), 25-hydroxyvitamin D_3_ (25-OH-VD_3_, Cat. No. HY-NE100), and osteocalcin (BGP, Cat. No. HY-J0001) were measured using ELISA kits (Beijing Huaying Institute of Biotechnology, Beijing, China).

#### Tibial indices

On day 49, one hen per replicate was randomly chosen, euthanized, and sampled. The left and right tibiae and femur were dissected, and all surrounding soft tissues and muscles were removed from the specimens. The following parameters were subsequently measured: tibial strength, fresh weight of the tibiae and femur, three-dimensional reconstruction using micro-computed tomography (micro-CT), calcium and phosphorus content.

The left tibia was used for both Micro-CT scanning and bone-strength testing. Scanning was performed on a MILabs U-CT-OI imaging system (MILabs B.V., Houten, the Netherlands) with the following parameters: tube voltage 55 kVp, tube current 0.17 mA, exposure time 75 ms, rotation step 0.357° over a total of 360°, and isotropic voxel resolution of approximately 35 μm. After scanning, three-dimensional reconstruction was performed using MILabs Rec v10.16 software. A region of interest (ROI) of 2.0 mm in height was selected starting 1.0 mm below the proximal growth plate of the tibia for trabecular bone analysis. The following bone microstructural parameters were calculated using IMALYTICS Preclinical v2.1 software (Gremse-IT GmbH, Aachen, Germany) according to the standardized nomenclature recommended by the American Society for Bone and Mineral Research (Bouxsein et al., 2010): bone volume fraction (BV/TV, %), trabecular thickness (Tb.Th, μm), trabecular separation (Tb.Sp, μm), and bone mineral density (BMD, mg/cm^3^).

Bone strength was measured using a texture analyzer (Stable Micro Systems Ltd., Godalming, UK) with a three-point bending test. The span was set at 40 mm, and the load cell was 1, 000 N. Tibiae were compressed at a descending speed of 0.2 mm/s until fracture. After strength measurement, tibial fragments were collected for subsequent analysis. Calcium and phosphorus contents were determined using inductively coupled plasma optical emission spectrometry (ICP-OES) according to the National Food Safety Standard for the Determination of Multiple Elements in Foods (GB 5009.268-2025).

#### Intestinal morphology

After euthanasia, the duodenum, jejunum, and ileum were separated from the colon. A 2 to 3 cm segment was cut from the middle of each intestinal section and fixed in 4% paraformaldehyde for 48 hours. Standard histological processing was performed, including washing, dehydration, clearing, paraffin-embedding, sectioning, and mounting. Sections were incubated in sequence with dewaxing solutions I and II (20 min each), absolute ethanol I and II (5 min each), and 75% ethanol (5 min). After a water wash, pre-staining solution was applied for 1 min. Hematoxylin staining lasted 3 to 5 min, followed by a water wash and H&E counterstaining. For each intestinal segment (duodenum, jejunum, and ileum) of each hen, four consecutive sections (50 μm apart) were prepared. At 40 × magnification, three complete and well-oriented villus-crypt units were randomly selected per section, yielding 15 measurements per segment. Villus height (VH) and crypt depth (CD) were measured using Image-Pro Plus 7.0 software, and the VH: CD ratio was calculated. All image analyses were performed by personnel blinded to treatment allocation (blinded analysis).

### Cecal content 16S rRNA microbiota analysis

#### Sequencing sample preparation

Fecal samples were obtained from 16 aged laying hens (control, n = 8; 0.5 mg/kg PQQ·Na_2_-treated, n = 8). The abdominal cavity was opened using a sterile scalpel, and the entire intestine was excised. The cecum was ligated at both ends, excised, and the outer surface was rinsed with PBS. Cecal contents were scraped using a sterile scalpel, aliquoted into 2 mL cryotubes, snap-frozen in liquid nitrogen, and stored at -80°C until 16S rRNA sequencing.

#### DNA extraction and PCR amplification

Genomic DNA was extracted from the samples using the HiPure Fecal DNA Kit (Magen) according to the manufacturer's instructions. DNA concentration and purity were detected using a NanoDrop 2000 and agarose gel electrophoresis, and the extracted DNA was stored at -20°C. Using the extracted genomic DNA as a template, the bacterial 16S rRNA gene was amplified with barcoded specific primers and Takara Ex Taq high-fidelity enzyme. Primers 343F and 798R were used to amplify the V3-V4 region for bacterial diversity analysis.

#### Library construction and sequencing

PCR-amplified products were detected using agarose gel electrophoresis. The products were purified using AMPure XP magnetic beads and used as the template for the second-round PCR. After a second round of purification using magnetic beads, the purified product was quantified using a Qubit fluorometer, and the concentration was adjusted for sequencing. Sequencing was performed using the Illumina NovaSeq 6000 sequencing platform (250 bp paired-end reads) by Shanghai OE Biotechnology Co., Ltd. (Shanghai, China).

#### Biological informatics analysis

Library construction, sequencing, and data analysis were completed by Shanghai OE Biomedical Technology Co., Ltd. Raw FASTQ data were downloaded, and primer sequences were removed using Cutadapt. Paired-end reads were processed using DADA2 with the default QIIME 2 parameters, including filtering, denoising, merging, and chimera removal. After quality control, representative sequences and an ASV abundance table were obtained. Representative ASV sequences were aligned against the SILVA database (version 138) for taxonomic annotation using q2-feature-classifier with default parameters. Alpha and beta diversity analyses were performed in QIIME 2. Alpha diversity was assessed using the Chao1 and Shannon indices. Principal coordinate analysis (PCoA) based on the unweighted UniFrac distance matrix was conducted in R to assess beta diversity. Differential abundance was evaluated using appropriate statistical tests, including ANOVA, Kruskal-Wallis, t-test, Wilcoxon rank-sum test, and LEfSe, as applicable.

#### Correlation analysis

To explore the associations between cecal microbiota and host physiological parameters, Spearman's rank correlation coefficients were calculated between dominant bacterial genera (relative abundance > 1%) and production performance as well as calcium and phosphorus metabolism parameters. Correlation analysis was performed using the corrplot package in R software (version 4.2.0). A correlation heatmap was generated to visualize the strength and significance of correlations between bacterial genera and each physiological parameter (*P* < 0.05). In the heatmap, red indicates positive correlation, blue indicates negative correlation, and the color intensity represents the absolute value of the correlation coefficient.

### Statistical analysis

All data were analyzed using SPSS 26.0 (IBM, Armonk, NY, USA). Before the formal experiment, one-way ANOVA confirmed that weekly average laying rate did not differ among groups during the 7-d pretrial period (*P* > 0.05). Experimental data were analyzed by one-way ANOVA, and multiple comparisons were performed using Duncan's multiple range test unless otherwise specified. Orthogonal polynomial contrasts were used to test linear and quadratic responses to increasing PQQ·Na_2_ supplementation levels (0.5, 1.0, 1.5, and 2.0 mg/kg). Results are presented as means ± SEM. Differences were considered significant at P < 0.05, and 0.05 ≤ P < 0.10 was considered a trend. 16S rRNA sequencing data and correlation analyses were performed on the OECloud platform (cloud.oebiotech.com).

## Results

### Effects of PQQ·Na_2_ on production performance of aged laying hens

The effects of PQQ·Na_2_ on the performance of aged laying hens are presented in Table 2. Compared with the control group, 0.5 mg/kg PQQ·Na_2_ treatment significantly increased the rate of laying during weeks 3 to 7 and throughout the entire experimental period (weeks 1 to 7) (*P* < 0.05). Specifically, during weeks 1 to 7, both 0.5 mg/kg and 1.0 mg/kg PQQ·Na_2_ treatments exhibited significantly higher laying rates than those in the control group (*P* < 0.05). No significant differences in average egg weight were observed between the control and treatment groups at any stage of the experiment (*P* > 0.05). Regarding feed intake, only the 2.0 mg/kg PQQ·Na_2_ treatment significantly decreased feed intake during weeks 1 to 3 compared with that of the control group (*P* < 0.05). Throughout the experimental period, no significant differences in the feed-to-egg ratio were observed among any of the treatment groups compared to that of the control group (*P* > 0.05).

**Table 2:** Effect of PQQ·Na_2_ on performance of aged laying hens.

Parameter	Control	PQQ.Na_2_, mg/kg				SEM	*P-*value
		0.5	1.0	1.5	2.0		
Laying rate, %							
1-3w	91.7	94.1	93.5	92.5	92.7	0.340	0.202
3-7w	91.1^a^	94.9^b^	92.8^a^	91.5^a^	91.3^a^	0.380	0.003
1-7w	91.3^a^	94.3^c^	93.4^bc^	91.6^ab^	92.4^abc^	0.340	0.013
Egg weight, g							
1-3w	62.6	62.3	62.5	62.2	63.2	0.200	0.414
3-7w	63.8	64.1	64.1	63.9	64.3	0.110	0.641
1-7w	63.2	63.3	63.4	63.2	63.8	0.160	0.708
1ADFI, g/d							
1-3w	116^b^	116^ab^	116^b^	116^b^	115^a^	0.160	0.036
3-7w	114	113	116	115	115	0.350	0.124
1-7w	115	114	116	115	115	0.230	0.090
Feed:egg ratio							
1-3w	1.97	2.04	1.99	2.05	2.09	0.020	0.339
3-7w	1.94	1.94	1.92	1.95	2.00	0.010	0.132
1-7w	1.95	1.98	1.95	1.99	2.04	0.010	0.134

1 ADFI = average daily feed intake. *n=8. Different superscript letters within the same row indicate significant differences (*P*< 0.05), while the same or no superscript letters indicate no significant differences (*P* > 0.05). SEM = standard error of the mean.

### Effects of PQQ.Na_2_ on egg quality of aged laying hens

The effects of PQQ·Na_2_ on the egg quality of aged laying hens are presented in Table 3. On day 20, no significant differences in the egg shape index were observed between the control and treatment groups (*P* > 0.05). On day 49, the 2.0 mg/kg PQQ·Na_2_ treatment exhibited a significantly lower egg shape index than that of the other groups (*P* < 0.05). Regarding eggshell strength, on both day 20 and day 49, the 0.5 mg/kg PQQ·Na_2_ treatment showed a significant increase compared with the control group (*P* > 0.05). On day 20, the 2.0 mg/kg PQQ·Na_2_ treatment did not differ significantly from the control (*P* > 0.05), whereas on day 49, it was significantly higher than that in the control (*P* < 0.05). Albumen height differed significantly on day 49. The 0.5 mg/kg PQQ·Na_2_ treatment exhibited significantly higher albumen height than the control and other treatment groups (*P* < 0.05). Compared with the control group, the 1.5 mg/kg PQQ·Na_2_ treatment showed an increase, but the difference was not significant (*P* > 0.05). No significant differences in Haugh units were observed between the control and treatment groups (*P* > 0.05). Yolk color was also not significantly affected by the supplementation level of PQQ·Na_2_ (*P* > 0.05).

**Table 3:** Effect of PQQ·Na_2_ on egg quality of aged laying hens.

Parameter	Control	PQQ.Na_2_, mg/kg				SEM	*P-*value
		0.5	1.0	1.5	2.0		
Egg shape index							
d 20	1.33	1.32	1.33	1.34	1.33	0.002	0.285
d 49	1.32^b^	1.32^b^	1.32^b^	1.34^b^	1.30^a^	0.003	0.003
Eggshell strength, N							
d 20	3.86^a^	4.38^b^	4.14^ab^	4.08^ab^	3.96^a^	0.060	0.044
d 49	3.84^a^	4.35^b^	4.16^ab^	4.17^ab^	4.22^b^	0.060	0.041
Albumen height, mm							
d 20	7.74	7.90	7.69	7.70	7.81	0.060	0.705
d 49	7.64^a^	8.25^b^	7.60^a^	8.03^ab^	7.51^a^	0.090	0.042
Haugh unit							
d 20	86.7	86.6	87.3	87.8	88.3	0.380	0.634
d 49	85.4	87.4	85.3	87.8	85.8	0.440	0.208
Yolk color							
d 20	6.88	7.00	6.94	6.75	6.88	0.050	0.670
d 49	7.13	7.00	7.13	7.13	7.38	0.060	0.414

*n=8. Different superscript letters within the same row indicate significant differences (*P*< 0.05), while the same or no superscript letters indicate no significant differences (*P* > 0.05). SEM = standard error of the mean.

### Effects of PQQ.Na_2_ on serum biochemical parameters of aged laying hens

The effects of PQQ·Na_2_ on the serum biochemical parameters of aged laying hens are presented in Table 4. Compared with the control group, alkaline phosphatase (ALP) activity was significantly increased in the 0.5 mg/kg PQQ·Na_2_ treated group (*P* < 0.05), indicating enhanced bone metabolism. Significant differences were observed in calcitonin (CT) and parathyroid hormone (PTH) levels across all treatment groups (*P* < 0.001). Specifically, calcitonin levels were significantly decreased in the 0.5 mg/kg PQQ·Na_2_ treated group compared to the control group. No significant differences in phosphorus concentration were observed among the groups (*P* > 0.05). Compared with the control group, 25-OH-VD_3_ concentration was significantly increased in the 0.5 mg/kg PQQ·Na_2_ treated group (*P* < 0.001), and osteocalcin (OCN) level was also significantly elevated in this group (*P* < 0.001).

**Table 4:** Effects of PQQ·Na_2_ on serum biochemical indexes in aged laying hens.

Parameter	Control	PQQ.Na_2_, mg/kg				SEM	*P-*value
		0.5	1.0	1.5	2.0		
ALP, U/L	133^a^	185^c^	152^ab^	162^bc^	172^bc^	5.500	0.003
CT, pg/mL	103^e^	76.7^a^	109^d^	85.7^b^	91.3^c^	3.760	< 0.001
PTH, pg/mL	109^d^	69.8^a^	86.9^c^	73.0^a^	85.0^b^	3.130	< 0.001
25⁃OH⁃VD_3_, ng/mL	21.9^a^	45.5^c^	25.5^ab^	30.0^b^	26.2^ab^	1.520	< 0.001
BGP, ng/mL	3.95^a^	5.28^c^	3.95^a^	4.62^b^	4.20^a^	0.110	< 0.001

*n=8. Different superscript letters within the same row indicate significant differences (*P*< 0.05), while the same or no superscript letters indicate no significant differences (*P* > 0.05). SEM = standard error of the mean. Alkaline Phosphatase (ALP); Calcitonin (CT); Parathyroid Hormone (PTH); 25-Hydroxyvitamin-D_3_(25-OH-VD_3_); Osteocalcin (BGP)

### Effects of PQQ.Na_2_ on calcium and phosphorus contents in serum and tibia of aged laying hens

The effects of PQQ·Na_2_ on calcium and phosphorus levels in the serum and tibia of aged laying hens are shown in Table 5. Compared with the control group, the serum calcium concentration was significantly increased in the 0.5 mg/kg PQQ·Na_2_ treated group (*P* < 0.05). Serum phosphorus concentrations showed no significant differences among any of the groups (*P* > 0.05); however, the 0.5 mg/kg PQQ·Na_2_ treatment group exhibited an increasing trend (0.47 mmol/L) compared with the control group (0.4 mmol/L).

**Table 5:** Effect of PQQ·Na_2_ on calcium and phosphorus content in serum and tibia of aged laying hens.

Parameter	Control	PQQ.Na_2_, mg/kg				SEM	*P-*value
		0.5	1.0	1.5	2.0		
Serum Calcium, mmol/L	5.80^a^	6.68^b^	5.86^a^	6.14^ab^	5.69^a^	0.110	0.038
Phosphorus, mmol/L	0.41	0.47	0.44	0.45	0.44	0.010	0.220
Tibia Calcium, %	2.82	2.85	2.88	2.83	2.85	0.010	0.692
Phosphorus, %	1.63	1.65	1.66	1.63	1.64	0.010	0.698

*n=8. Different superscript letters within the same row indicate significant differences (*P*< 0.05), while the same or no superscript letters indicate no significant differences (*P* > 0.05). SEM = standard error of the mean.

### Effects of PQQ.Na_2_ on tibial quality of aged laying hens

The effects of PQQ·Na_2_ on tibia quality in aged laying hens are presented in Fig. 6. Dietary supplementation with PQQ·Na_2_ significantly affected bone mineral density (BMD) and bone volume fraction (BV/TV) in laying hens compared to the control group. BMD exhibited a trend of initially increasing and then decreasing with increasing PQQ·Na_2_ supplementation. The 0.5 mg/kg PQQ·Na_2_ treatment group showed a significant increase in BMD compared to the control group (*P* < 0.05). The BV/TV trend was consistent with that of the BMD. The 0.5 mg/kg PQQ·Na_2_ treatment group exhibited significantly higher BV/TV than the control and other treatment groups (*P* < 0.001). No significant differences in BV/TV were observed between the other treatment and control groups (*P* > 0.05). Regarding bone microarchitecture, no significant differences in trabecular thickness (Tb.Th) or trabecular separation (Tb.Sp) were observed between any treatment group and the control group (*P* > 0.05). However, the 0.5 mg/kg PQQ·Na_2_ treatment group showed a decreasing trend in Tb.Sp (454.22 μm) compared with the control group (577.52 μm). No significant differences in tibial weight, length, or strength were observed between the control and treatment groups (*P* > 0.05). Notably, tibial strength showed an increasing trend in the 0.5 mg/kg (220.75 N) and 1.0 mg/kg (207.18 N) PQQ·Na_2_ treatment groups compared with that in the control group (178.94 N) (Table 6).

**Table 6:** Effect of PQQ·Na_2_ on tibia of aged laying hens.

Parameter	Control	PQQ.Na_2_, mg/kg				SEM	*P-*value
		0.5	1.0	1.5	2.0		
BMD, mg/cm^3^	1.26^a^	1.44^b^	1.31^a^	1.28^a^	1.27^a^	0.026	0.020
BV/TV, %	37.3^a^	43.5^b^	38.2^a^	34.9^a^	35.3^a^	0.970	< 0.001
Tb.Th, μm	214	222	224	226	228	4.42	0.785
Tb.Sp, μm	579	454	590	604	622	41.3	0.550
Tibial Weight, g	5.47	5.94	6.00	5.34	5.78	0.230	0.222
Tibial Length, cm	7.94	8.04	8.06	7.78	8.00	0.300	0.367
Tibial Strength, N	179	221	207	193	178	8.86	0.095

*n=8. Different superscript letters within the same row indicate significant differences (*P*< 0.05), while the same or no superscript letters indicate no significant differences (*P* > 0.05). SEM = standard error of the mean. Bone Mineral Density (BMD); Bone Volume/Total Volume (BV/TV); Trabecular Thickness (Tb.Th); Trabecular Separation (Tb.Sp)

### Effects of PQQ.Na_2_ on intestinal morphology of aged laying hens

**Fig. 1:**
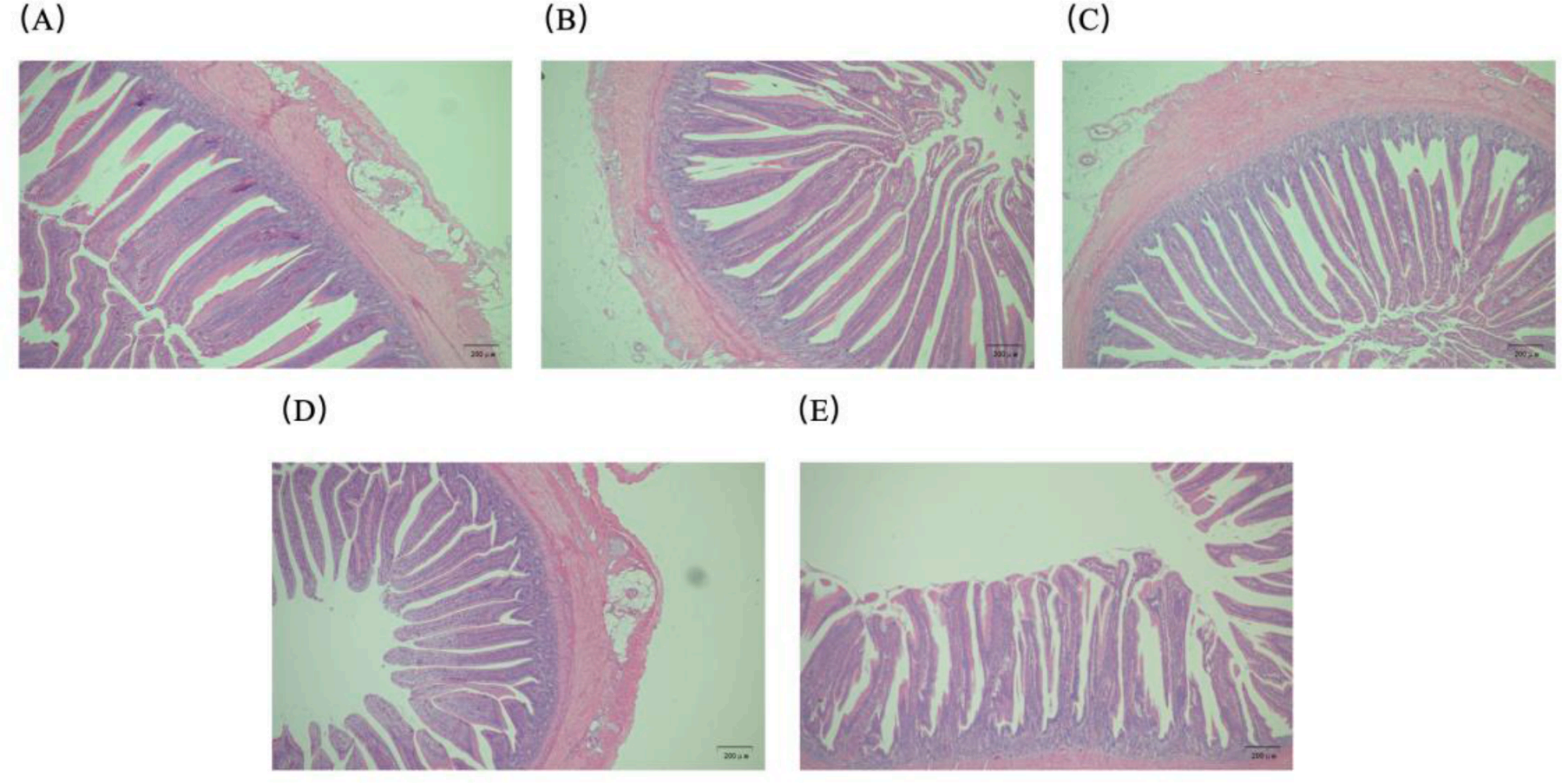
Effects of PQQ.Na_2_ on intestinal morphology in aged laying hens. Representative H&E-stained sections of the ileum are shown for (A) control group; (B) 0.5 mg/kg PQQ.Na_2_ group; (C) 1.0 mg/kg PQQ.Na_2_ group; (D) 1.5 mg/kg PQQ.Na_2_ group; (E) 2.0 mg/kg PQQ.Na_2_ group. Scale bar in (A) = 200 μm (applies to all panels).

Fig. 1 shows the effects of PQQ.Na_2_ supplementation on the ileal intestinal structure of aged hens. Hematoxylin and eosin (H&E) staining (Fig. 1 A–E) corresponded to the control and the group supplemented with 0.5, 1.0, 1.5, and 2.0 mg/kg PQQ.Na_2_, respectively. Compared with the control group, the villus height-to-crypt depth ratio showed an increasing trend in the 0.5 mg/kg PQQ.Na_2_ group.

The effects of PQQ·Na_2_ on the intestinal morphology of aged laying hens are shown in Table 7. Dietary supplementation with PQQ·Na_2_ exerted differential effects on morphological parameters across different intestinal segments in laying hens. In the jejunum, compared with the control group, the 0.5 mg/kg PQQ·Na_2_ treatment group exhibited significantly increased villus height and crypt depth (*P* < 0.05), whereas no significant differences were observed between the other treatment groups and the control group (*P >* 0.05).In the ileum, compared with the control group, the 0.5 mg/kg PQQ·Na_2_ treatment group showed a significant decrease in crypt depth (*P* < 0.05) and a tendency for an increased villus height-to-crypt depth ratio (V/C ratio) (P = 0.083).In the duodenum, no significant differences were observed in any of the morphological parameters across the treatment groups (*P* > 0.05).

**Table 7:** Effect of PQQ·Na_2_ on intestinal morphology of aged laying hens.

Parameter	Control	PQQ.Na_2_, mg/kg				SEM	*P-*value
		0.5	1.0	1.5	2.0		
Jejunum VH, μm	1481	1841	1555	1555	1543	57.0	0.310
CD, μm	199	215	218	197	202	8.70	0.932
VH:CD ratio	7.7	8.56	7.30	7.97	7.65	0.200	0.357
Ileum VH, μm	1346	1284	1387	1384	1271	34.7	0.773
CD, μm	196^b^	155^a^	197^b^	184^b^	168^ab^	5.12	0.019
VH:CD ratio	6.98	8.32	7.09	7.54	7.54	0.180	0.083
Duodenum VH, μm	2232	2345	2135	2133	2324	36.2	0.183
CD, μm	283	292	301.	294	311	8.08	0.874
VH:CD ratio	8.00	8.10	7.17	7.28	7.57	0.200	0.532

*n=8. Different superscript letters within the same row indicate significant differences (*P*< 0.05), while the same or no superscript letters indicate no significant differences (*P* > 0.05). SEM = standard error of the mean. Villus Height (VH); Crypt Depth (CD); Villus Height to Depth Ratio (VH/CD)

### 16S rRNA sequencing of cecal content microbiota

#### Effect of PQQ.Na_2_ on cecal microbiota in aged laying hens

Samples for 16S rRNA sequencing were selected using a stratified validation design. After evaluating phenotypic indicators across all five treatment groups, the 0.5 mg/kg PQQ·Na_2_ group exhibited the greatest improvements in laying rate, eggshell strength, serum calcium, and bone mineral density, and was thus identified as the optimal supplementation level. Following a "broad screening followed by focused investigation" multi-omics approach, 16S rRNA sequencing was performed on the control group (n = 8) and the optimal dose group (n = 8) to investigate the gut microbiota-mediated mechanisms underlying the beneficial effects of PQQ·Na_2_ on calcium-phosphorus metabolism and bone health in aged laying hens. This two-group comparison ensured sufficient statistical power while avoiding resource redundancy associated with full-gradient sequencing.

#### Sequencing data quality assessment

To investigate the effect of PQQ·Na_2_ on the cecal microbiota structure of aged laying hens, cecal content samples from 16 aged laying hens in the 0.5 mg/kg PQQ·Na_2_ group and the control group (n = 8 per group) were selected for 16S rRNA V3-V4 region sequencing. Sequencing was performed on an Illumina NovaSeq 6000 platform with 250-bp paired-end reads. A total of 78,033 to 81,646 raw reads were obtained per sample. After filtering, 38,814 to 48,049 clean reads were retained.

#### Cecal microbiota OTUs analysis

**Fig. 2:**
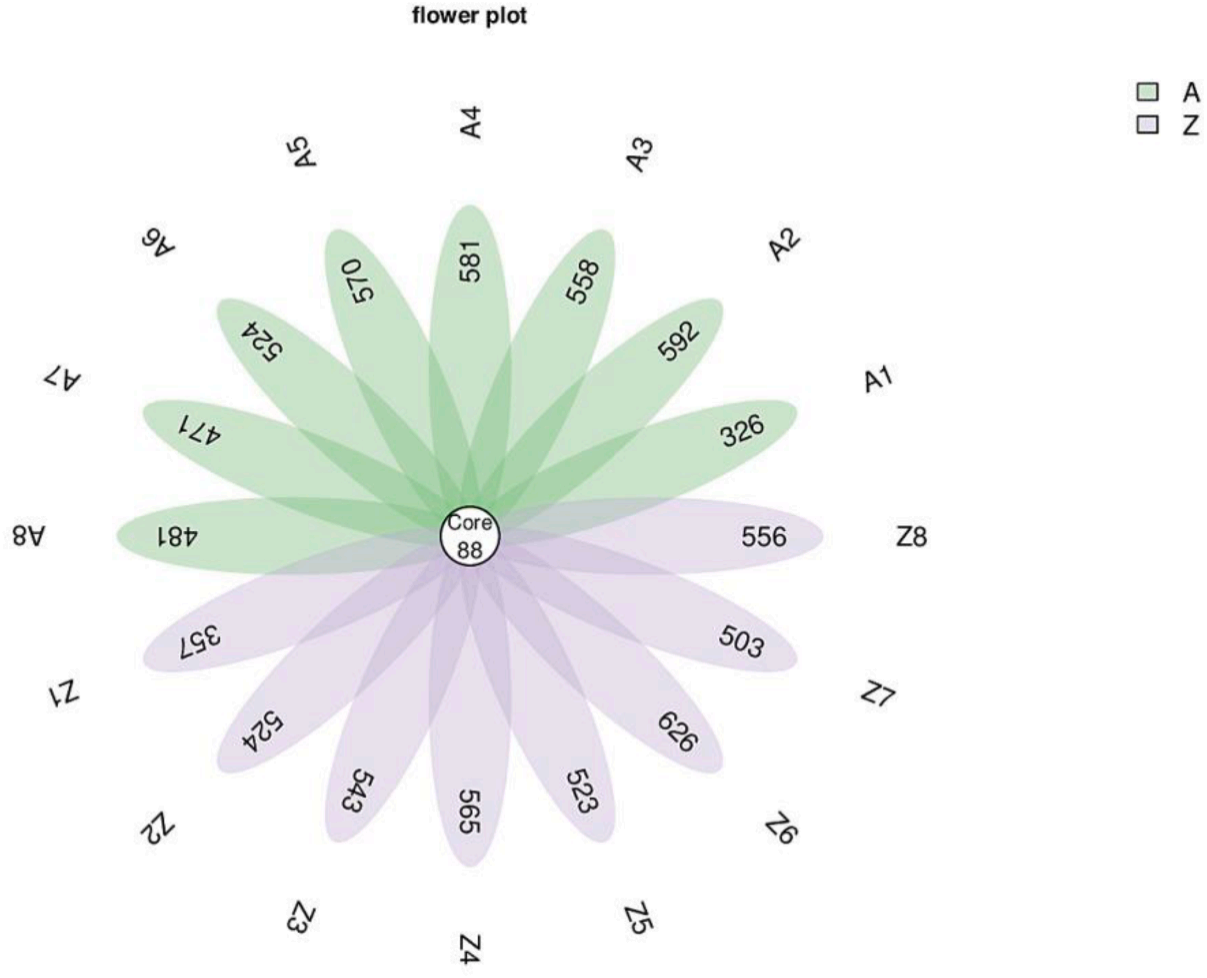
Sample-Based Core OTUs Petal Plot.

Fig. 2 presents the sample-based core OTUs petal plot. Based on cluster analysis at 97% similarity, 88 core OTUs were identified across all samples, indicating the presence of a shared core microbiota in the cecum of aged laying hens in both the control and 0.5 mg/kg PQQ·Na_2_ groups.

### Alpha diversity analysis

#### Alpha diversity analysis

**Fig. 3:**
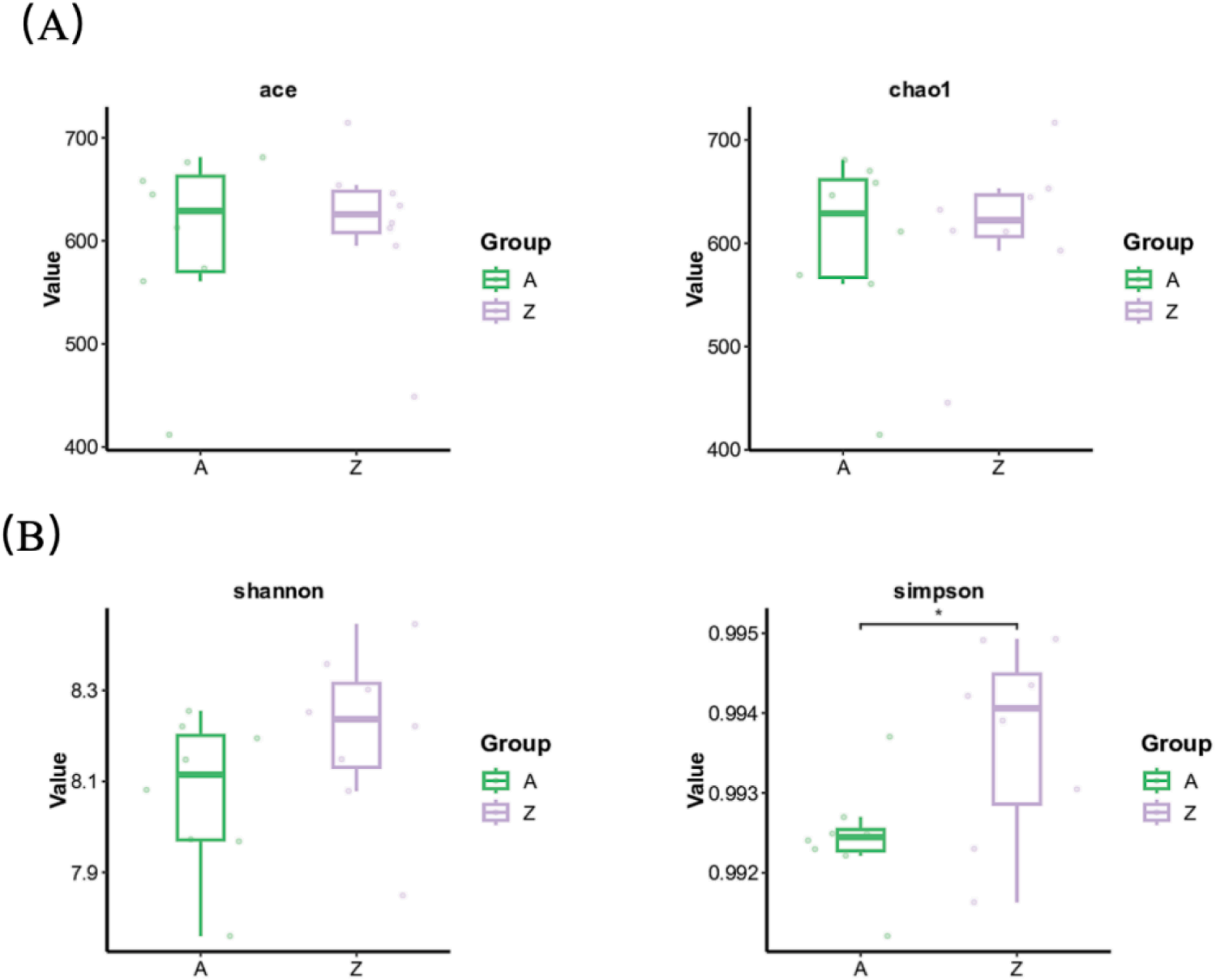
Effects of PQQ·Na_2_ supplementation on microbial community diversity (Shannon index) (A) and richness (Chao1 index) (B) in cecal contents.

Fig. 3 illustrates the microbial community diversity (Shannon index, A) and richness (Chao1 index, B) in cecal contents following PQQ·Na_2_ supplementation. Because the 0.5 mg/kg PQQ·Na_2_ group exhibited the most pronounced improvements across multiple indicators, cecal content samples from the control group and the 0.5 mg/kg PQQ·Na_2_ group were selected for 16S rRNA gene sequencing to investigate the effect of PQQ·Na_2_ on the intestinal microbiota structure of aged laying hens.

#### Species diversity analysis

**Fig. 4:**
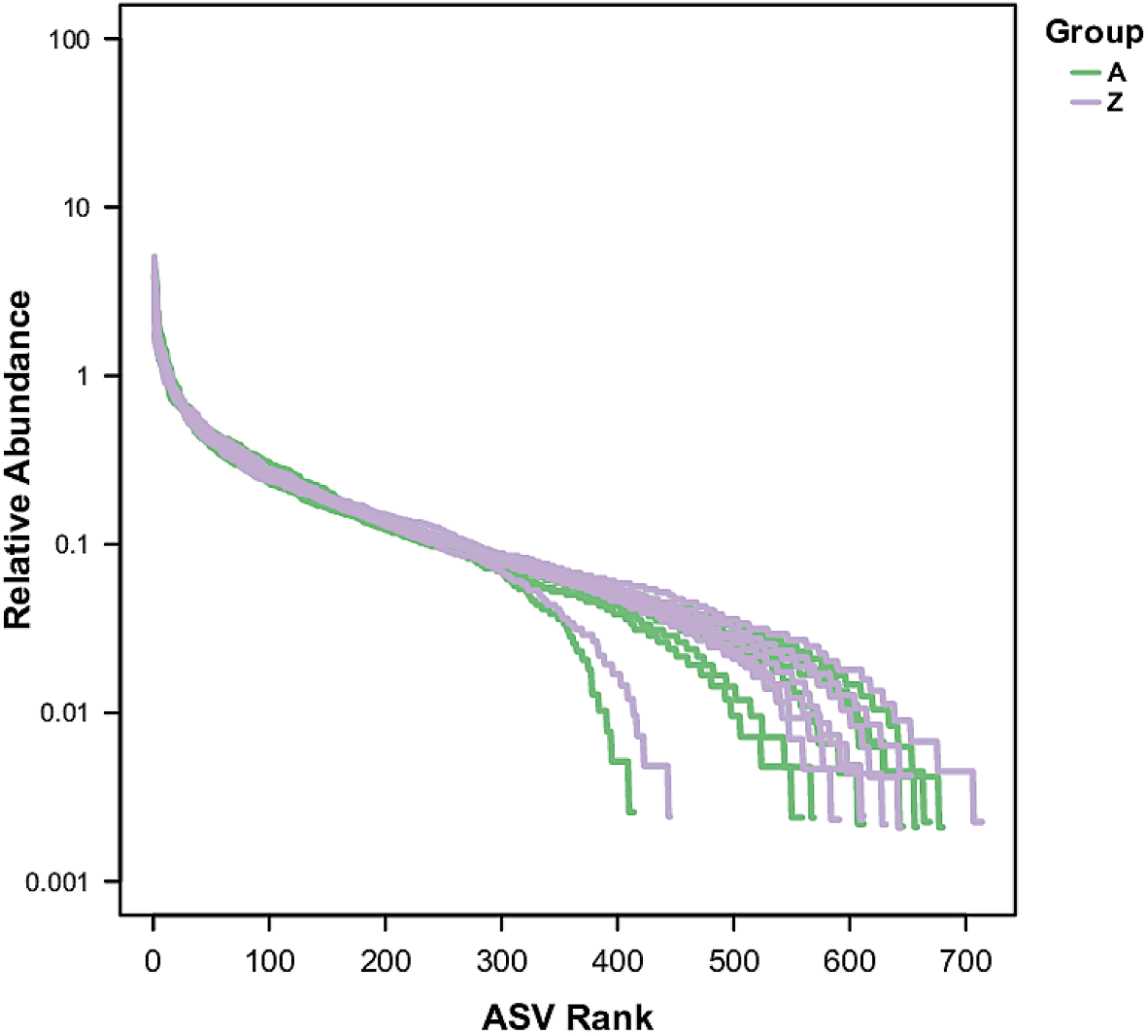
Rank-abundance Curve Analysis.

Fig. 4 shows the rank-abundance curve analysis used to evaluate species diversity. The analysis of species richness and evenness using the rank-abundance curve revealed that the curve covered a broad range on the horizontal axis (including 88 core OTUs), indicating high species richness in the samples.

#### Species accumulation curve

**Fig. 5:**
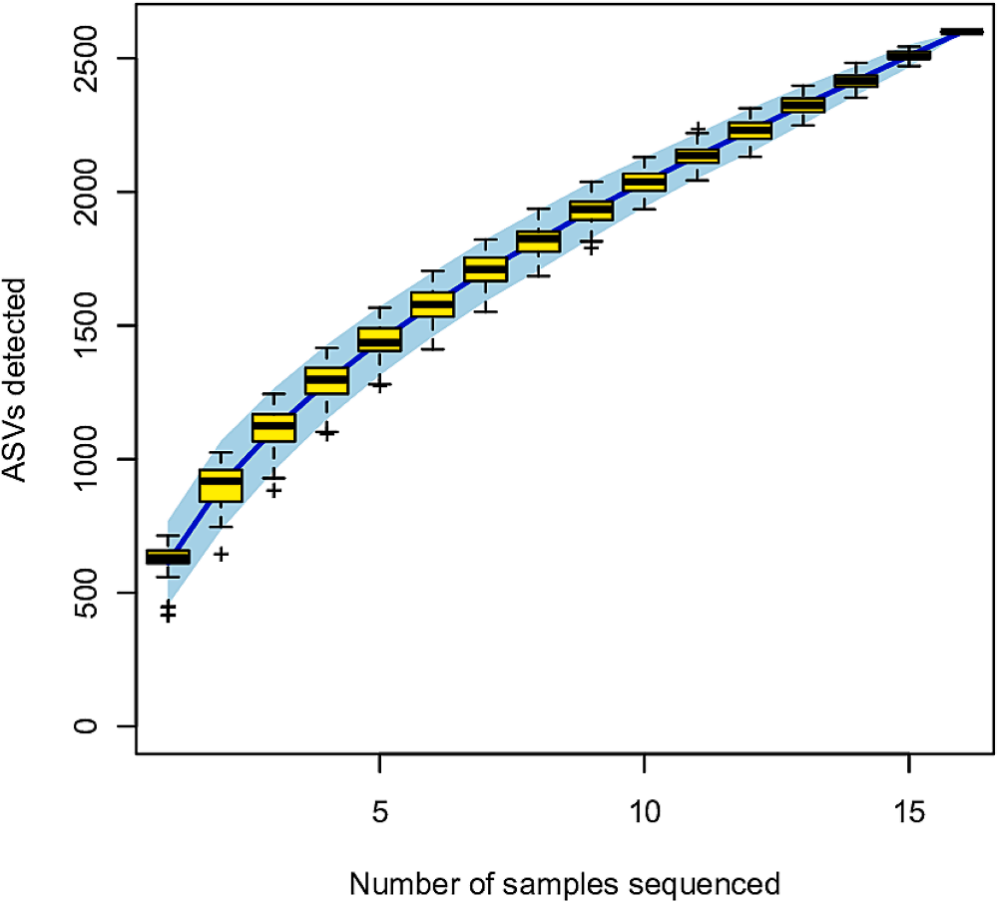
Species Accumulation Curve.

Fig. 5 shows the species accumulation curve analysis of microbial communities. Based on cluster analysis with 97% similarity, a total of 88 core OTUs were identified across all samples, indicating that 88 core microbial communities were shared in the cecum of aged laying hens between the control group and the 0.5 mg/kg PQQ·Na_2_ group.

#### Microbial community analysis

**Fig. 6:**
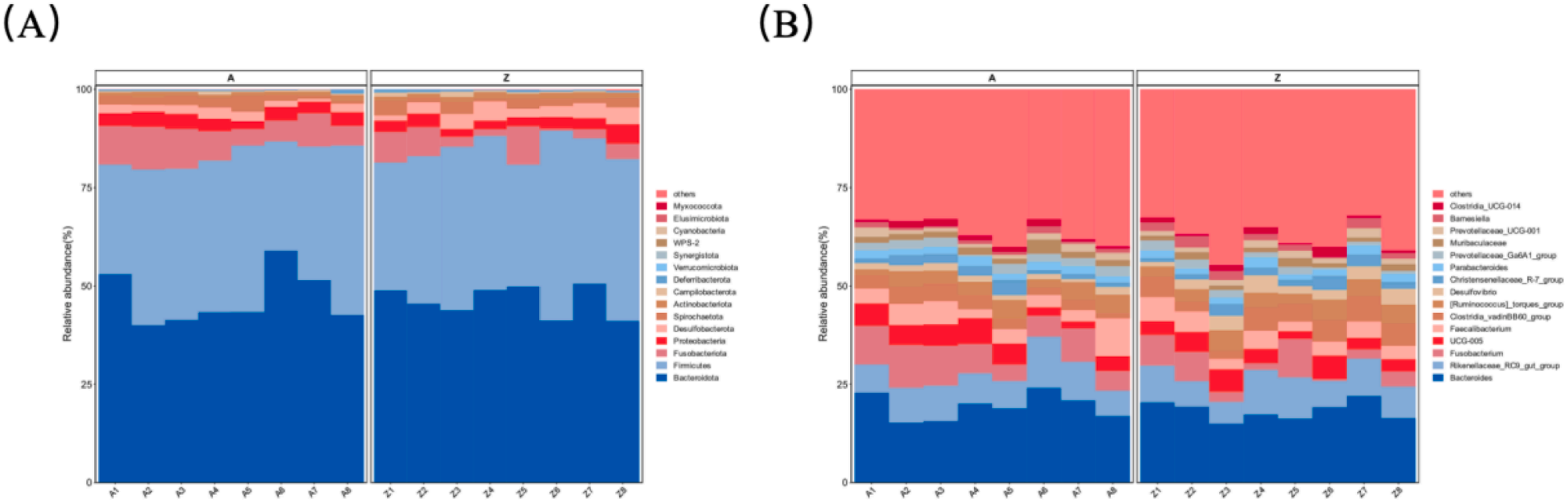
(A) Abundance of the top 10 dominant microbes at the phylum level, and (B) abundance of the top 10 dominant microbes at the genus level.

Fig. 6 shows the abundance of the top 10 dominant microorganisms at the phylum and genus levels in the cecal microbiota of the control and experimental groups.

At the phylum level, the cecal microbiota in both groups was dominated by Bacteroidetes and Firmicutes, with their combined relative abundances accounting for more than 80% of the total microbiota, followed by Spirochaetota. The relative abundances of Bacteroidota in the 0.5 mg/kg PQQ.Na_2_ group and the control group were 47% and 46%, respectively; the abundances of Firmicutes were 36% and 39%, respectively; and the abundances of Spirochaetota were 2% and 2%, respectively.

At the genus level, *Bacteroides* was the dominant genus in both groups, with relative abundances of 19.3% and 18.3% in the 0.5 mg/kg PQQ·Na_2_ group and the control group, respectively. The next most abundant genus was *Rikenellaceae_RC9_gut_group*, with relative abundances of 8.3% and 8.4%, respectively, followed by *Fusobacterium*, with relative abundances of 7.2% and 4.5%, respectively.

#### LEfSe analysis of differential microbiota

**Fig. 7:**
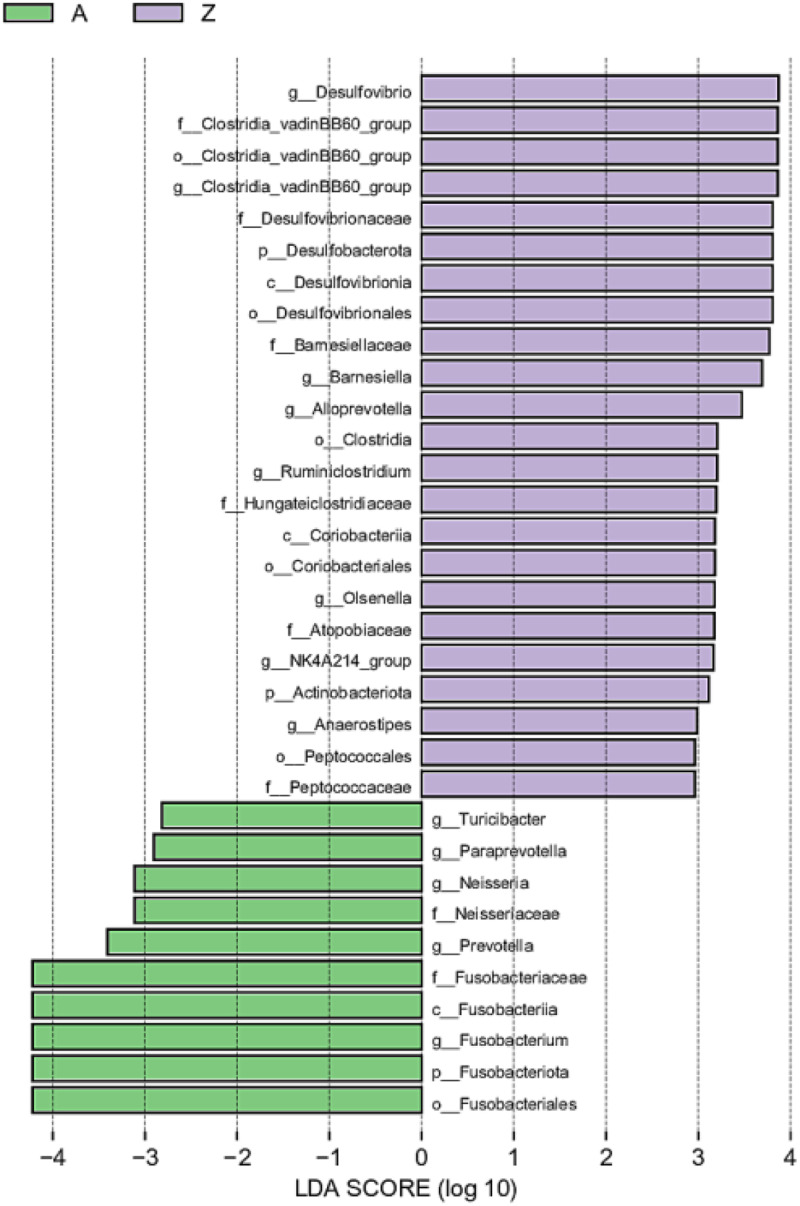
LEfSe analysis.

Fig. 7 shows the differential microbial biomarkers between the two groups, screened using LEfSe analysis. The results indicated differences in microbial community structure between the two groups at multiple taxonomic levels.

At the phylum level, *Desulfobacterota* was significantly enriched in the control group, whereas no phylum was significantly enriched in the 0.5 mg/kg PQQ·Na_2_ group.

At the genus level, the control group was enriched with *Clostridium_vadinBB50_group, Barnesiella, Alloprevotella.* In the 0.5 mg/kg PQQ·Na_2_ group, the enriched microorganisms mainly included *Prevotella, Neisseria, Turicibacter*, and *Fusobacterium.*

#### Analysis of microbial functional changes

**Fig. 8:**
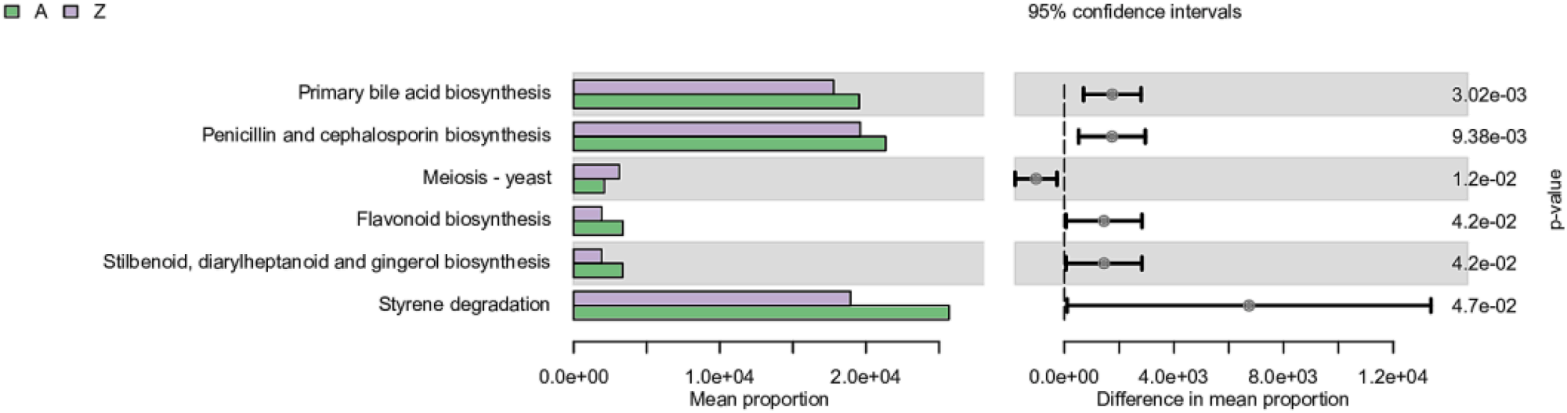
Statistical chart of KEGG pathway abundance.

Fig. 8 shows the KEGG pathway abundance statistics. Based on the PICRUSt2 functional prediction, the relative microbial abundance in each sample was analyzed at KEGG pathway levels 1 and 2. The results showed that differential microorganisms were mainly enriched in several metabolic pathways. The significantly enriched pathways included flavonoid biosynthesis, stilbenoid, diarylheptanoid, and gingerol biosynthesis, penicillin and cephalosporin biosynthesis, styrene degradation (related to xenobiotic degradation and metabolism), primary bile acid biosynthesis (associated with host-microbe co-metabolism); and meiosis (related to cellular processes) (*P* < 0.05).

## Discussion

In this study, dietary supplementation with 0.5 mg/kg PQQ·Na_2_ significantly improved the laying rate of aged laying hens, with stable performance observed during the mid and late experimental periods. As a novel coenzyme, PQQ·Na_2_ may enhance mitochondrial function, energy metabolism, and lipid metabolism, thereby alleviating the decline in reproductive function caused by oxidative stress in aged laying hens (Qiu et al., 2021; Long et al., 2022; Charrier et al., 2024). These findings are consistent with previous reports demonstrating that PQQ·Na_2_ improves animal production performance (Zhang et al., 2025).

No significant differences were observed in average egg weight or feed-to-egg ratio among treatment groups, suggesting that the improvement in laying performance induced by PQQ·Na_2_ was mainly attributable to an increased laying rate. Eggshell strength, a critical indicator of egg quality during transport and storage, was significantly increased in the 0.5 mg/kg PQQ·Na_2_ group on both days 20 and 49, suggesting that PQQ·Na_2_ may improve eggshell quality by modulating calcium and phosphorus metabolism or shell gland function. Albumen height was also significantly increased in the 0.5 mg/kg PQQ·Na_2_ group on day 49, indicating a beneficial effect on albumen quality that may be associated with the antioxidant properties of PQQ·Na_2_ (Wang et al., 2018; Shao et al., 2023). The egg shape index was significantly reduced only in the 2.0 mg/kg PQQ·Na_2_ group on day 49, suggesting a dose-specific effect on egg shape; however, the underlying mechanism requires further investigation. Haugh unit and yolk color did not differ significantly among groups, indicating that PQQ·Na_2_ had limited effects on albumen viscosity and yolk pigmentation (Zhang et al., 2016).

Serum biochemical parameters collectively delineated a multi-hormonal synergistic profile of PQQ·Na_2_ in reestablishing calcium-phosphorus homeostasis. In the present study, serum alkaline phosphatase (ALP) and osteocalcin (BGP) levels were significantly increased in the 0.5 mg/kg PQQ·Na_2_ group, indicating enhanced osteoblastic activity. Meanwhile, calcitonin (CT) and parathyroid hormone (PTH) levels were significantly decreased, reflecting a physiological state in which blood calcium is sufficient due to improved intestinal absorption efficiency, thereby obviating the need for extensive bone calcium mobilization (Wu et al., 2017; Huang et al., 2018). Furthermore, Xue et al. (2026) demonstrated that PQQ activates the Nrf2 signaling pathway and transcriptionally upregulates parathyroid hormone receptor (PTH1R) expression via the Nrf2-Pth1r axis, thereby potentiating the osteogenic effects of PTH and enhancing peak bone mass. This finding suggests that even under conditions of reduced circulating PTH levels, PQQ·Na_2_ may enhance PTH sensitivity at the receptor level, contributing synergistically to the observed improvements in bone- and eggshell-related calcium metabolism (Xue et al., 2026).

Notably, 25-OH-VD_3_ levels were significantly elevated in this group. Kuwata et al. (2024) demonstrated that the 25(OH)D_3_-DBP complex is taken up by renal proximal tubular cells via megalin- and cubilin-mediated endocytosis and subsequently converted to active 1,25(OH)_2_D_3_. The age-related decline in cubilin expression suppresses this process, leading to deteriorated eggshell quality in aged laying hens (Kuwata et al., 2024). In the present study, PQQ·Na_2_ may indirectly promote vitamin D activation via 25-hydroxylase by improving hepatic mitochondrial function. This hypothesis is supported by Stites et al. (2006), who reported that PQQ deficiency led to a 20-30% reduction in hepatic mitochondrial content in mice, while PQQ supplementation stimulated mitochondrial complex I activity (Stites et al., 2006). Furthermore, Qiu et al. (2021) demonstrated that PQQ·Na_2_ treatment significantly increased mitochondrial DNA content and upregulated the expression of mitochondrial biogenesis-related genes (TFAM, PGC-1α, and NRF-1) in hepatocytes.

Serum biochemical parameters reflect metabolic status and bone metabolic activity. In this study, the activity of alkaline phosphatase (ALP), a marker of osteoblast activity, was significantly increased in the 0.5 mg/kg PQQ·Na_2_ group, indicating enhanced bone metabolism (Swetha et al., 2025). Calcitonin (CT) and parathyroid hormone (PTH) levels were significantly decreased in the 0.5 mg/kg PQQ·Na_2_ group, suggesting that PQQ·Na_2_ may regulate calcium and phosphorus metabolism by inhibiting bone resorption and promoting bone formation (Pechet et al., 1967). The levels of 25-hydroxyvitamin D_3_ and osteocalcin were significantly increased in the 0.5 mg/kg PQQ·Na_2_ group, further supporting the positive regulatory role of PQQ·Na_2_ in bone metabolism. Elevated vitamin D_3_ levels facilitate calcium absorption and deposition, whereas increased osteocalcin levels reflect enhanced osteoblast function (Verlinden and Carmeliet, 2021). Collectively, these results indicate that an appropriate dosage of PQQ·Na_2_ (0.5 mg/kg) promotes bone health in aged laying hens by modulating the hormones involved in calcium metabolism.

Serum calcium content was significantly increased in the 0.5 mg/kg PQQ·Na_2_ group, suggesting that PQQ·Na_2_ may promote calcium absorption and transport, which is consistent with the elevated serum 25-hydroxyvitamin D_3_ levels in this group. This positive association aligns with the findings of Qiu et al. (2021), who demonstrated that PQQ supplementation in laying hens enhanced hepatic mitochondrial biogenesis, thereby potentially supporting the hepatic 25-hydroxylation of vitamin D. Furthermore, Kuwata et al. (2024) established that the 25(OH)D_3_-DBP complex undergoes megalin/cubilin-mediated endocytosis in renal proximal tubules for activation to 1,25(OH)_2_D_3_, a process that declines with age in laying hens (Qiu et al., 2021; Kuwata et al., 2024).Although serum phosphorus levels did not differ significantly among the groups, an increasing trend was observed in the 0.5 mg/kg PQQ·Na_2_ group, potentially reflecting the coordinated regulation of calcium and phosphorus metabolism. The tibial calcium and phosphorus contents did not differ significantly among the groups, suggesting that PQQ.Na_2_ exerts a relatively mild effect on bone mineralization. This relatively mild effect is consistent with previous observations that PQQ primarily acts as a modulator of osteoblastic activity and calciotropic hormone signaling rather than directly promoting bone mineral deposition(Wu et al., 2017; Xue et al., 2026).

Regarding tibial quality, both bone mineral density (BMD) and bone volume fraction (BV/TV) were significantly increased in the 0.5 mg/kg PQQ·Na_2_ group, exhibiting a trend of initial increase followed by a decrease, indicating a dose-dependent improvement in bone microarchitecture by PQQ·Na_2_ (Li et al., 2023). These findings align with Geng et al. (2019), who demonstrated that PQQ prevented ovariectomy-induced bone loss and improved bone microarchitecture by inhibiting oxidative stress and osteocyte senescence (Geng et al., 2019). Trabecular separation (Tb.Sp) showed a decreasing trend in this group, suggesting a more compact trabecular structure, further supporting the beneficial effects of PQQ·Na_2_ on bone quality. Although tibial strength did not reach statistical significance, both the 0.5 mg/kg and 1.0 mg/kg PQQ·Na_2_ groups showed a clear increasing trend compared with the control group, indicating that PQQ·Na_2_ may enhance bone mechanical properties to some extent. Consistent with this observation, Geng et al. (2019) also reported that PQQ treatment significantly improved bone strength in osteoporotic mice, with effects comparable to those of estrogen (Geng et al., 2019).

In summary, PQQ·Na_2_ improves bone quality in aged laying hens, with an optimal supplemental dose of 0.5 mg/kg. In this study, the 0.5 mg/kg PQQ·Na_2_ group exhibited significantly increased jejunal villus height and crypt depth, significantly decreased ileal crypt depth, and an increasing trend in the villus height-to-crypt depth ratio, indicating that PQQ·Na_2_ may promote intestinal development, enhance absorptive surface area, and improve nutrient absorption efficiency (Yin et al., 2019). Increased villus height expands the intestinal absorptive surface area and enhances nutrient absorption efficiency, whereas decreased crypt depth reflects accelerated intestinal turnover, which helps maintain mucosal barrier integrity(Huang et al., 2020). No significant changes were observed in the duodenal parameters, which may be related to the differential sensitivity of intestinal segments to PQQ·Na_2_. Overall, PQQ·Na_2_ improved intestinal morphology in aged laying hens, particularly in the jejunum and ileum, increasing the intestinal absorptive surface area and enhancing the absorption efficiency of Ca and P. This may represent one of the key mechanisms by which PQQ·Na_2_ improves production performance and calcium-phosphorus metabolism(Yin et al., 2019).

In this study, 16S rRNA gene sequencing revealed that the 0.5 mg/kg PQQ·Na_2_ group exhibited increasing trends in the Ace and Chao1 indices and a significant decrease in the Simpson index (*P* < 0.05), indicating that PQQ·Na_2_ increased the species richness of the cecal microbiota and altered microbial diversity (Liu et al., 2023). Similar results have been reported in other animal models, where PQQ supplementation promoted the recovery of gut microbiota diversity and reconstructed the microbiota composition under pathological conditions (Xu et al., 2025). Increased gut microbiota diversity is generally associated with improved host health and is beneficial for maintaining intestinal barrier function and metabolic homeostasis(John et al., 2025).

At the phylum level, the dominant phyla in both groups were *Bacteroidota* and *Firmicutes*, with their combined abundance exceeding 80% of the total microbiota, which is consistent with the typical characteristics of the poultry gut microbiota (Oakley et al., 2014). LEfSe analysis showed that *Desulfobacterota* was significantly enriched in the control group. *Desulfobacterota* are sulfate-reducing bacteria; their metabolite, hydrogen sulfide (H_2_S), at high concentrations, inhibits tissue cellular respiration, leading to intestinal epithelial cell damage and impaired intestinal barrier function (Linden, 2014). Intestinal barrier integrity is crucial for calcium and phosphorus absorption, and intestinal epithelial damage leads to decreased expression of calcium-binding protein (CaBP), thereby reducing active calcium transport and absorption efficiency(Hu et al., 2022). Therefore, the inhibitory effect of PQQ·Na_2_ on *Desulfobacterota* may indirectly promote intestinal calcium absorption by preserving intestinal barrier integrity.

At the genus level, the control group was enriched in *Clostridium_vadinBB50_group, Barnesiella*, and other bacteria, whereas the PQQ·Na_2_ group was enriched in *Prevotella, Turicibacter*, and other bacteria. *Prevotella* is closely associated with the production of short-chain fatty acids (SCFAs) in the intestine. SCFAs (particularly butyrate) can lower the intestinal pH and increase the solubility of calcium and phosphorus, thereby enhancing the calcium and phosphorus absorption efficiency (Barone et al., 2022). In addition, SCFAs can regulate the proliferation and differentiation of intestinal epithelial cells and enhance calcium-binding protein expression by activating G protein-coupled receptors (e.g., GPR41 and GPR43) (Samuel et al., 2008). Therefore, the promotion of SCFA-producing bacteria, such as *Prevotella*, by PQQ·Na_2_ may represent an important mechanism by which it improves calcium and phosphorus metabolism. In this study, the abundance of *Fusobacterium* in the PQQ·Na_2_ group (7.2%) was higher than that in the control group (4.5%). *Fusobacterium* is a genus of opportunistic pathogens that also exists in low abundance in healthy intestines (Li et al., 2025). The observed increase in *Fusobacterium* abundance in the PQQ·Na_2_ group may represent either a direct effect of PQQ·Na_2_ on the microbial community or an indirect consequence of improved intestinal environment, and its functional significance remains to be elucidated

Functional prediction analysis based on PICRUSt2 showed that the differential microorganisms between the PQQ.Na_2_ and control groups were mainly enriched in multiple metabolic pathways. Among them, the significant enrichment of pathways such as flavonoid biosynthesis and stilbenoid biosynthesis suggested that PQQ·Na_2_ may affect the secondary metabolite synthesis capacity of cecal microbiota. Flavonoids are natural products with antioxidant, anti-inflammatory, and immunomodulatory properties. They regulate bone metabolism by inhibiting osteoclast activity and promoting osteoblast differentiation. Flavonoids can also modulate the gut microbiota structure and indirectly affect calcium absorption(Du et al., 2024). The enrichment of the primary bile acid biosynthesis pathway indicates that PQQ·Na_2_ may be involved in regulating host-microbe co-metabolism. This is consistent with findings that PQQ supplementation improves liver biological functions and enhances lipid metabolism, thereby potentially influencing bile acid synthesis (Qiu et al., 2021). The amphipathic structure of bile acids promotes the emulsification, digestion, and absorption of fat-soluble vitamins (including vitamin D), and bile acids are also associated with the vitamin D receptor (VDR) (Šarenac and Mikov, 2018). Vitamin D is a core regulator of calcium and phosphorus metabolism, promoting intestinal calcium absorption and renal tubular calcium reabsorption. The microbiota may enhance vitamin D absorption and utilization by regulating bile acid metabolism, thereby improving calcium and phosphorus metabolism.

The enrichment of penicillin and cephalosporin biosynthesis pathways suggests that PQQ·Na_2_ may affect the activity of certain antibiotic-producing bacteria in the cecal microbiota. These functional prediction results provide new perspectives for understanding the molecular mechanisms by which PQQ·Na_2_ regulates intestinal health in aged laying hens; however, the specific mechanisms still need further validation through metabolomics and other approaches.

Notably, there were no significant differences in tibial calcium and phosphorus content among the groups. This result suggests that although PQQ·Na_2_ promoted improvements in bone mineral density and microstructure, its effect on the basal mineralization status of bone was relatively mild. Combined with the increased serum calcium and 25-hydroxyvitamin D_3_ levels as well as enhanced ALP activity observed in this study, it can be inferred that PQQ·Na_2_ may primarily increase bone mineral density by promoting medullary bone formation and enhancing trabecular bone connectivity, rather than by altering the basal mineralization quality of cortical or trabecular bone. This phenomenon holds important physiological significance in high-yielding laying hens. Enhancing bone function by optimizing bone microstructure while maintaining stable bone mineralization quality may represent a potential mechanism by which PQQ·Na_2_ regulates bone metabolism in aged laying hens.

In summary, dietary supplementation with 0.5 mg/kg PQQ·Na_2_ improves laying performance, eggshell quality, and bone health in aged laying hens through multiple pathways, including the regulation of calcium-phosphorus metabolism-related hormones, promotion of bone microstructure optimization, and modulation of cecal microbiota composition. In particular, the finding that tibial calcium and phosphorus contents showed no significant change while bone mineral density significantly increased suggests that PQQ·Na_2_ may enhance bone function by increasing trabecular bone compactness and promoting medullary bone hyperplasia without altering the basal bone mineralization quality. This mode of action is of great significance for maintaining bone health in older laying hens. Future studies should further investigate the specific molecular mechanisms underlying the effects of PQQ·Na_2_ on the interaction between bone metabolism and gut microbiota.

## Ethics approval

All animal procedures were approved by the Animal Care Advisory Committee of Zhejiang University (ZJU20260328, Hangzhou, China). The birds were handled in accordance with the guidelines for the care and use of laboratory animals, and all efforts were made to minimize suffering.

### Declaration of generative AI and AI-assisted technologies in the writing process

During the preparation of this work, the authors used ChatGPT (OpenAI) for grammar checking and language editing. After using this tool, the authors reviewed and edited the content as needed and take full responsibility for the content of the publication.

### Funding

This study was funded by the Xinchang Tianmu Laboratory Research Fund (TM2023008). The funder had no role in study design, data collection, analysis, interpretation, or manuscript preparation.

## CRediT authorship contribution statement

**M. Feng:** Writing – review & editing, Writing – original draft, Validation, Data curation. **Y. Tang:** Investigation. **Q. Huang:** Resources. **M. Lu:** Formal analysis. **H. Zeng:** Investigation. **Y. Bai:** Funding acquisition. **J. Lu:** Conceptualization.

## Disclosures

The authors declare that they have no known competing financial interests or personal relationships that could have appeared to influence the work reported in this paper.

## References

[bib0001] Aboulhassane S., Sangha V., Bendayan R. (2026). Protective effects of pyrroloquinoline quinone in CNS disorders. J. Nutr. Biochem..

[bib0002] Barone M., D'Amico F., Brigidi P., Turroni S. (2022). Gut microbiome-micronutrient interaction: The key to controlling the bioavailability of minerals and vitamins?. BioFactors.

[bib0003] Bouxsein M.L., Boyd S.K., Christiansen B.A., Guldberg R.E., Jepsen K.J., Müller R. (2010). Guidelines for assessment of bone microstructure in rodents using micro-computed tomography. J. Bone Miner. Res..

[bib0004] Charrier D., Cerullo G., Carpenito R., Vindigni V., Bassetto F., Simoni L., Moro T., Paoli A. (2024). Metabolic and biochemical effects of Pyrroloquinoline Quinone (PQQ) on inflammation and mitochondrial dysfunction: potential health benefits in obesity and future perspectives. Antioxidants (Basel).

[bib0005] Chowanadisai W., Bauerly K.A., Tchaparian E., Wong A., Cortopassi G.A., Rucker R.B. (2010). Pyrroloquinoline Quinone stimulates mitochondrial biogenesis through cAMP response element-binding protein phosphorylation and increased PGC-1α expression. J. Biol. Chem.

[bib0006] Cohen J. (2013).

[bib0008] Du J., Wang Y., Wu C., Zhang X., Zhang X., Xu X. (2024). Targeting bone homeostasis regulation: potential of traditional Chinese medicine flavonoids in the treatment of osteoporosis. Front. Pharmacol..

[bib0007] European Commission (2018). Commission Implementing Regulation (EU) 2018/1122 of 10 August 2018 authorising the placing on the market of pyrroloquinoline quinone disodium salt as a novel food pursuant to Regulation (EC) No 258/97. Off. J. Eur. Union L.

[bib0009] Garcia-Mejia R.A., Sinclair-Black M., Blair L.R., Angel R., Jaramillo B., Regmi P., Neupane N., Proszkowiec-Weglarz M., Arbe X., Cavero D., Ellestad L.E. (2024). Physiological changes in the regulation of calcium and phosphorus utilization that occur after the onset of egg production in commercial laying hens. Front. Physiol..

[bib0010] Geng Q., Gao H., Yang R., Guo K., Miao D. (2019). Pyrroloquinoline quinone prevents estrogen deficiency-induced osteoporosis by inhibiting oxidative stress and osteocyte senescence. Int. J. Biol. Sci..

[bib0011] Hester P.Y., Hester P.Y. (2017). Egg Innovations and Strategies for Improvements.

[bib0012] Hu Y.X., van Baal J., Hendriks W.H., Duijster M., van Krimpen M.M., Bikker P. (2022). Mucosal expression of Ca and P transporters and claudins in the small intestine of broilers is altered by dietary Ca:P in a limestone particle size dependent manner. PLoS One.

[bib0013] Huang C., Ming D., Wang W., Wang Z., Hu Y., Ma X., Wang F. (2020). Pyrroloquinoline Quinone alleviates jejunal mucosal barrier function damage and regulates colonic microbiota in piglets challenged with enterotoxigenic *Escherichia coli*. Front. Microbiol..

[bib0014] Huang Y., Chen N., Miao D. (2018). Pyrroloquinoline quinone plays an important role in rescuing Bmi-1(-/-) mice induced developmental disorders of teeth and mandible–anti-oxidant effect of pyrroloquinoline quinone. Am. J. Transl. Res..

[bib0015] John H.T., Thomas T.C., Chukwuebuka E.C., Ali A.B., Anass R., Tefera Y.Y., Babu B., Negrut N., Ferician A., Marian P. (2025). The microbiota-human health axis. Microorganisms.

[bib0016] Kuwata N., Mukohda H., Uchida H., Takamatsu R., Binici M.M., Yamada T., Sugiyama T. (2024). Renal Endocytic Regulation of Vitamin D Metabolism during Maturation and Aging in Laying Hens. Animals (Basel).

[bib0017] Lay D.C., Fulton R.M., Hester P.Y., Karcher D.M., Kjaer J.B., Mench J.A., Mullens B.A., Newberry R.C., Nicol C.J., O’Sullivan N.P., Porter R.E. (2011). Hen welfare in different housing systems. Poult. Sci..

[bib0018] Li J., Zhang J., Xue Q., Liu B., Qin R., Li Y., Qiu Y., Wang R., Goltzman D., Miao D., Yang R. (2023). Pyrroloquinoline quinone alleviates natural aging-related osteoporosis via a novel MCM3-Keap1-Nrf2 axis-mediated stress response and Fbn1 upregulation. Aging Cell.

[bib0019] Li Z., Liu J., Li J., Zhou Z., Huang X., Gopinath D., Luo P., Wang Q., Shan D. (2025). Fusobacterium in the microbiome: from health to disease across the oral–gut axis and beyond. npj Biofilms Microbiomes.

[bib0020] Linden D.R. (2014). Hydrogen sulfide signaling in the gastrointestinal tract. Antioxid. Redox Signal..

[bib0021] Liu X., Jiang W., Lu G., Qiao T., Gao D., Zhang M., Cai H., Chai L., Yi W., Lv Z. (2023). The Potential Role of Pyrroloquinoline Quinone to Regulate Thyroid Function and Gut Microbiota Composition of Graves' Disease in Mice. Pol. J. Microbiol..

[bib0022] Long C., Wang Z., Guo Y., Sheng X., Xing K., Ni H., Wang X., Xiao L., Qi X. (2022). Research Note: Dietary supplementation with pyrroloquinoline quinone disodium (PQQ.Na(2)) improves oxidative status and semen quality in aging layer breeder roosters. Poult. Sci..

[bib0023] National Health Commission of the People's Republic of China (2022).

[bib0024] Oakley B.B., Lillehoj H.S., Kogut M.H., Kim W.K., Maurer J.J., Pedroso A., Lee M.D., Collett S.R., Johnson T.J., Cox N.A. (2014). The chicken gastrointestinal microbiome. FEMS Microbiol. Lett..

[bib0025] Pechet M.M., Bobadilla E., Carroll E.L., Hesse R.H. (1967). Regulation of bone resorption and formation. Influences of thyrocalcitonin, parathyroid hormone, neutral phosphate and vitamin D3. Am. J. Med..

[bib0026] Petricca S., Matrone A., Capece D., Flati I., Flati V., Ricevuto E., Celenza G., Franceschini N., Mastrangelo M., Pellegrini C., Cristiano L., Familiari G., Cinque B., Di Emidio G., Tatone C., Iorio R. (2025). Pyrroloquinoline Quinone (PQQ) Attenuates Hydrogen Peroxide-Induced Injury Through the Enhancement of Mitochondrial Function in Human Trabecular Meshwork Cells. Int. J. Mol. Sci..

[bib0027] Qiu K., Zhao Q., Wang J., Qi G.H., Wu S.G., Zhang H.J. (2021). Effects of pyrroloquinoline quinone on lipid metabolism and anti-oxidative capacity in a high-fat-diet metabolic dysfunction-associated fatty liver disease chick model. Int. J. Mol. Sci..

[bib0028] Salisbury S.A., Forrest H.S., Cruse W.B., Kennard O. (1979). A novel coenzyme from bacterial primary alcohol dehydrogenases. Nature.

[bib0029] Samuel B.S., Shaito A., Motoike T., Rey F.E., Backhed F., Manchester J.K., Hammer R.E., Williams S.C., Crowley J., Yanagisawa M., Gordon J.I. (2008). Effects of the gut microbiota on host adiposity are modulated by the short-chain fatty-acid binding G protein-coupled receptor, Gpr41. Proc. Natl. Acad. Sci. USA.

[bib0030] Šarenac T.M., Mikov M. (2018). Bile Acid Synthesis: From Nature to the Chemical Modification and Synthesis and Their Applications as Drugs and Nutrients. Front. Pharmacol..

[bib0031] Shao D., Liu L., Tong H., Shi S. (2023). Dietary pyrroloquinoline quinone improvement of the antioxidant capacity of laying hens and eggs are linked to the alteration of Nrf2/HO-1 pathway and gut microbiota. Food Chem. X.

[bib0032] Sinclair-Black M., Garcia R.A., Ellestad L.E. (2023). Physiological regulation of calcium and phosphorus utilization in laying hens. Front. Physiol..

[bib0033] Stites T., Storms D., Bauerly K., Mah J., Harris C., Fascetti A., Rogers Q., Tchaparian E., Satre M., Rucker R.B. (2006). Pyrroloquinoline quinone modulates mitochondrial quantity and function in mice. J. Nutr..

[bib0034] Swetha S., Rao G.N., Mahalakshmi V., Sathya R. (2025). Alkaline phosphatase and acid phosphatase in health and disease - A systematic review. J. Oral Maxillofac. Pathol..

[bib0035] Turck D., Bresson J.L., Burlingame B., Dean T., Fairweather-Tait S., Heinonen M., Hirsch-Ernst K.I., Mangelsdorf I., McArdle H.J., Naska A., Neuhäuser-Berthold M., Nowicka G., Pentieva K., Sanz Y., Siani A., Sjödin A., Stern M., Tomé D., Vinceti M., Willatts P., Engel K.H., Marchelli R., Pöting A., Poulsen M., Schlatter J.R., de Sesmaisons A., Van Loveren H. (2017). Safety of pyrroloquinoline quinone disodium salt as a novel food pursuant to Regulation (EC) No 258/97. EFSA J..

[bib0036] Verlinden L., Carmeliet G. (2021). Integrated View on the Role of Vitamin D Actions on Bone and Growth Plate Homeostasis. JBMR Plus.

[bib0037] Wan Y., Du Q., Wang D., Ma R., Qi R., Yang R., Li X., Li J., Liu W., Li Y., Zhan K. (2023). Effects of different-sized cages on the production performance, serum parameters, and Caecal microbiota composition of laying hens. Animals.

[bib0038] Wang J., Yang L.L., Zhang H.J., Wu S.G., Qi G.H. (2018). Effects of pyrroloquinoline quinone disodium on performance, antioxidant status and plasma biochemical parameters of laying hens. Chin. J. Anim. Nutr..

[bib0039] Wang J., Wang W.W., Qi G.H., Cui C.F., Wu S.G., Zhang H.J., Xu L., Wang J. (2021). Effects of dietary Bacillus subtilis supplementation and calcium levels on performance and eggshell quality of laying hens in the late phase of production. Poult. Sci..

[bib0040] Wu X., Li J., Zhang H., Wang H., Yin G., Miao D. (2017). Pyrroloquinoline quinone prevents testosterone deficiency-induced osteoporosis by stimulating osteoblastic bone formation and inhibiting osteoclastic bone resorption. Am. J. Transl. Res..

[bib0041] Xu H., Wang X., Peng W., Hu Y., Xu Y., Xiao X., Dai B., Zhang R., Zhong Y., Yang C. (2025). Dietary Pyrroloquinoline Quinone addition alleviated weaning stress via modulation of gut microbiota and metabolic profiles in weaned piglets. Animals (Basel).

[bib0042] Xue Q., Gu Y., Li J., Qin R., Li M., Li Y., Chen Z., Li C., Zhang J., Yang R. (2026). Nutritional activation of the Nrf2–Pth1r axis by pyrroloquinoline quinone enhances peak bone mass and potentiates teriparatide in osteoporosis. Free Radic. Biol. Med..

[bib0043] Yin X., Ming D., Bai L., Wu F., Liu H., Chen Y., Sun L., Wan Y., Thacker P.A., Wu G., Wang F. (2019). Effects of pyrroloquinoline quinone supplementation on growth performance and small intestine characteristics in weaned pigs. J. Anim. Sci..

[bib0044] Zhang Y., Wu S.G., Wang J. (2016). Effects of tea polyphenols, vitamin E and pyrroloquinoline quinone disodium on performance and antioxidant capacity of laying hens during late laying period. Chin. J. Anim. Sci..

[bib0045] Zhang Z., Gao Z., Jia Z. (2025). Pyrroloquinoline quinone promotes porcine oocyte in vitro maturation and subsequent embryo development by enhancing lipid metabolism and improving mitochondrial function. Anim. Biosci..

